# Deep groundwater irrigation altered microbial community and increased anammox and methane oxidation in paddy wetlands of Sanjiang Plain, China

**DOI:** 10.3389/fmicb.2024.1354279

**Published:** 2024-02-21

**Authors:** Huai Li, Aiwen Song, Ling Qiu, Shen Liang, Zifang Chi

**Affiliations:** ^1^State Key Laboratory of Black Soils Conservation and Utilization, Northeast Institute of Geography and Agroecology, Chinese Academy of Sciences, Changchun, China; ^2^University of Chinese Academy of Sciences, Beijing, China; ^3^Second Hospital of Jilin University, Changchun, China; ^4^Key Lab of Groundwater Resources and Environment, Ministry of Education, Jilin University, Changchun, China

**Keywords:** paddy wetlands, soil indigenous microorganisms, groundwater's irrigation, microbial community, microbial function

## Abstract

The over-utilizing of nitrogen fertilizers in paddy wetlands potentially threatens to the surrounding waterbody, and a deep understanding of the community and function of microorganisms is crucial for paddy non-point source pollution control. In this study, top soil samples (0–15 cm) of paddy wetlands under groundwater's irrigation at different depths (H1: 6.8 m, H2: 13.7 m, H3: 14.8 m, H4: 15.6 m, H5: 17.0 m, and H6: 17.8 m) were collected to investigate microbial community and function differences and their interrelation with soil properties. Results suggested some soil factor differences for groundwater's irrigation at different depths. Deep-groundwater's irrigation (H2-H6) was beneficial to the accumulation of various electron acceptors. Nitrifying-bacteria *Ellin6067* had high abundance under deep groundwater irrigation, which was consistent with its diverse metabolic capacity. Meanwhile, denitrifying bacteria had diverse distribution patterns. Iron-reducing bacteria *Geobacter* was abundant in H1, and *Anaeromyxobacter* was abundant under deep groundwater irrigation; both species could participate in Fe-anammox. Furthermore, *Geobacter* could perform dissimilatory nitrate reduction to ammonia using divalent iron and provide substrate supply for anammox. *Intrasporangium* and *norank_f_Gemmatimonadacea* had good chromium- and vanadium-reducting potentials and could promote the occurrence of anammox. Low abundances of methanotrophs *Methylocystis* and *norank_f_Methyloligellaceae* were associated with the relatively anoxic environment of paddy wetlands, and the presence of aerobic methane oxidation was favorable for *in-situ* methane abatement. Moisture, pH, and TP had crucial effects on microbial community under phylum- and genus-levels. Microorganisms under shallow groundwater irrigation were highly sensitive to environmental changes, and Fe-anammox, nitrification, and methane oxidation were favorable under deep groundwater irrigation. This study highlights the importance of comprehensively revealing the microbial community and function of paddy wetlands under groundwater's irrigation and reveals the underlying function of indigenous microorganisms in agricultural non-point pollution control and greenhouse gas abatement.

## Graphical Abstract

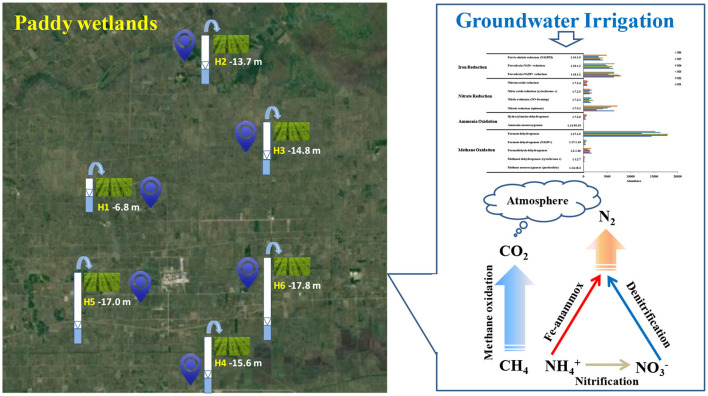

## Highlights

- Deep groundwater irrigation favors the accumulation of multi-electron acceptors
- *Geobacter* and *Anaeromyxobacter* could participate in Fe-anammox
- *Methylocystis* and *norank_f_Methyloligellaceae* involve *in-situ* abatement of methane
- Moisture, pH, and TP play important roles in shaping microbial community
- Deep groundwater irrigation promotes Fe-anammox, nitrification, and methane oxidation

## 1 Introduction

Paddy wetlands, in which the constructed wetlands were coupled with water and fertilizer, had typical wetland ecosystems and vegetation characteristics and could provide unique habitats for microorganisms to ensure global food security (Ding et al., 2021). However, the excessive nitrogen-fertilizers application under large-scale irrigation could cause serious soil degradation and surrounding-water pollution, posing a major risk to human's health (Sun et al., 2021). In addition, paddy wetlands were one of the major sources of methane emission (Sun et al., 2019). Soil microorganisms regulated wetland biogeochemical cycles (Torsvik and Øvreås, 2002). Microbial species had various functions, such as denitrification and methane oxidation (Li et al., 2014; Chi et al., 2021a). Some biotic or abiotic factors could control microbial community composition and function, and microorganisms were sensitive and could respond rapidly to environmental changes (Guo et al., 2019; Pérez Castro et al., 2019). Therefore, revealing the community and function of microorganisms in paddy wetlands was crucial for maintaining their ecological stability and material management.

As efficient agronomic measures, fertilization and irrigation could affect microbial community and diversity in paddy wetlands (Wang et al., 2021). Environmental changes obviously altered microbial communities and activities, thereby affecting biogeochemical processes. Long-term fertilization could alter soil physicochemical properties, thereby affecting microbial communities (Guo et al., 2020). Hou et al. (2022) found that the long-term application of pig manure could promote the enrichment of complex organic-degrading bacteria and improve soil enzyme activity. Inorganic fertilizers could increase microbial diversity and change microbial community composition in paddy wetlands (Huang et al., 2019). The type of fertilizer had minimal influence on microbial community under long-term fertilization (Liu et al., 2022). Furthermore, irrigation could ensure rice growth and affect microbial community. Jiang et al. (2021) showed that irrigation combined with biochar could alleviate greenhouse gas emissions and alter microbial community. Irrigation modes had some effects on microbial community of paddy wetlands (Jin et al., 2020). Reclaimed water irrigation could improve soil microbial activity and fertility to a certain extent (Wei et al., 2017). Although some studies investigated microbial community of paddy wetlands under fertilization and irrigation, the effects of groundwater's irrigation at different depths on microbial community and diversity in paddy wetlands remain unknown.

Recent studies were involved in the function and metabolic potential of microorganisms (Chi et al., 2021d). Li et al. (2019) revealed the responses of nitrogen-metabolized pathways and relevant function genes to coastal environmental variations. Exogenous pollution input could alter microbial community, thus changing their metabolic function (Chi et al., 2021b). The genes encoding exogenous metabolism and nitrate-reducting were significantly enriched on coastal sediments (Su et al., 2018). The Sanjiang Plain was regarded as a representative of large-scale paddy development, and paddy wetlands was mainly irrigated using groundwater with various electron acceptors (Cao et al., 2016; Chong et al., 2020; Meng et al., 2021; Chen et al., 2024). However, research on the community and function of microorganisms in paddy wetlands under groundwater's irrigation was still lacking.

Microorganisms in paddy wetlands had vital effects on the nitrogen cycle, but a large knowledge gap existed regarding the influence of groundwater irrigation. We hypothesized that groundwater irrigation had significant effects on microbial communities and functions of paddy wetlands, and that the input of exogenous electron acceptors could improve soil material transformation. To test this hypothesis, we analyzed soil genetic data from paddy wetlands under groundwater irrigation using high-throughput sequencing. The aims in this study were as follows: (1) to reveal the microbial community composition and difference in paddy wetlands under groundwater's irrigation at different depths, (2) to elucidate the responses in the metabolic-pathways and related-enzyme genes of microorganisms, and (3) to explore the dominant enablers involving in microbial community and functional variation in paddy wetlands. This study can offer valuable academic references for non-point source pollution control and greenhouse gas reduction of paddy wetlands.

## 2 Materials and methods

### 2.1 Sampling area description

**Figure 1:**
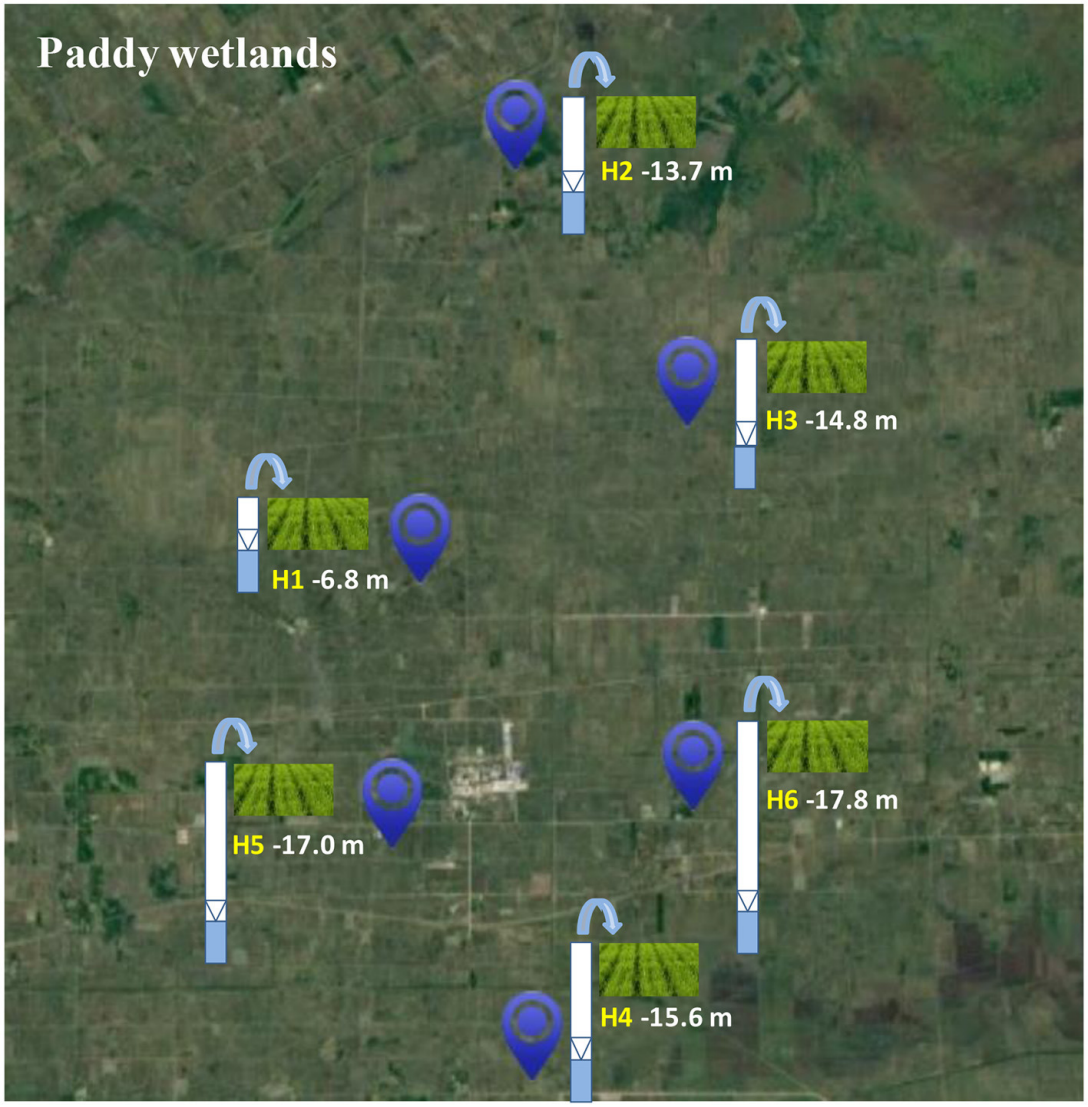
Sampling sites of paddy wetlands under groundwater irrigation.

The research area was situated on Honghe Farm of Sanjiang Plain in the Heilongjiang Province, China, which belonged to the temperate- and continental-monsoon climate. The mean temperature and rainfall per year were 1.6°C−1.9°C and 565–600 mm, respectively. Precipitation was concentrated from June to August. Furthermore, the growing period of rice was from May to September, and the type of the soil was paddy soil. Urea was the main fertilizer with an average fertilization rate of 180 kg N/hm^2^. The paddy wetlands in this area were mainly assarted from swamps and dry land in the last few decades, which were under groundwater's irrigation. Therefore, paddy wetlands under groundwater's irrigation at different depths, such as H1 (−6.8 m), H2 (−13.7 m), H3 (−14.8 m), H4 (−15.6 m), H5 (−17.0 m), and H6 (−17.8 m), were selected to investigate the underlying influence of groundwater's irrigation on microbial community and function of paddy wetlands (Figure 1). Here, we defined shallow- and deep-groundwater as less and more than 10 m at depth.

### 2.2 Soil collecting and analysis

Top soil samples (0–15 cm) in paddy wetlands were collected in triplicgot and mixed into a composite sample at sampling sites H1 (47°37′39^′′^-N; 133°29′11^′′^-E), H2 (47°43′15^′′^-N; 133°31′6^′′^-E), H3 (47°39′47^′′^-N; 133°34′35^′′^-E), H4 (47°30′55^′′^-N; 133°31′27^′′^-E), H5 (47°34′4^′′^-N; 133°28′37^′′^-E), and H6 (47°34′35^′′^-N; 133°34′41^′′^-E) in May 2021. Six soil samples were obtained and named H1, H2, H3, H4, H5, and H6. All soil samples were placed in plastic bags and then sent to the nearby laboratory on ice packs. A part was air-dried for 3 weeks at room-temperature and then filtered using a 2-mm sifter in order to determinate soil properties. And the rest part was stored in a −80°C in order to analyse microbial community and function. Soil physicochemical factors were tested as previously described (Li et al., 2019).

### 2.3 Illumina sequence and statistical analyses

Total DNA from the six soil samples was extracted by means of PowerSoil-DNA-isolation kit (MoBio, Carlsbad, CA). Moreover, V3-V4 region in 16S rRNA gene was amplifed using primers 338F and 806R. PCR amplification was performed in a total volume of 20 μL (contained 4 μL Buffer, 0.4 μL FastPfu Polymerase, 2 μL dNTP, 5 μM of each primer and 10 ng genome DNA). Thermal cycling conditions were as follows: an initial denaturation at 95°C for 3 min, followed by 35 cycles at 95°C for 30 s, 55°C for 30 s, and 72°C for 45 s, with a final extension at 72°C for 10 min. The PCR products were further performed based on Illumina-MiSeq-platform (Shanghai-Majorbio Bio-pharm Technology Co., Ltd., China). Processing of raw sequences obtained from Illumina sequencing was performed using QIIME software (version 1.9.1). We assembled paired-end reads using FLASH (version 1.2.11, https://ccb.jhu.edu/software/FLASH/index.shtml), where forward and reverse reads had overlapping base lengths ≥10 bp, and base mismatches were prevented. Reads with a quality score < 20, ambiguous bases, and improper primers were discarded before clustering. High-quality sequences of 252096 were acquired with 36934–48033 sequences for each soil sample. The obtained sequences were further clustered through USEARCH with 97% similarity as OTUs. And the taxonomy was verified by the Silva databases. To reduce the influence of sequencing depth on treatment effects, the samples were randomly resampled to the same sequence depth based on the least number of sequences. Correlation analysis (CA) was used to uncover the interrelation between crucial phyla, function gene, and soil property. Principal coordinate analysis (PCoA) was utilized to identify microbial community difference. Redundancy analysis was performed to investigate the key factors shaping microbial community and function. Shannon index, functional gene, and metabolic pathway were obtained using Quantitative Insights into Microbial Ecology (QIIME) and Phylogenetic Investigation of Communities by Reconstruction of Unobserved States (PICRUSt) (Chen et al., 2016; Guo et al., 2022).

## 3 Results and discussion

### 3.1 Soil characteristics

**Figure 2:**
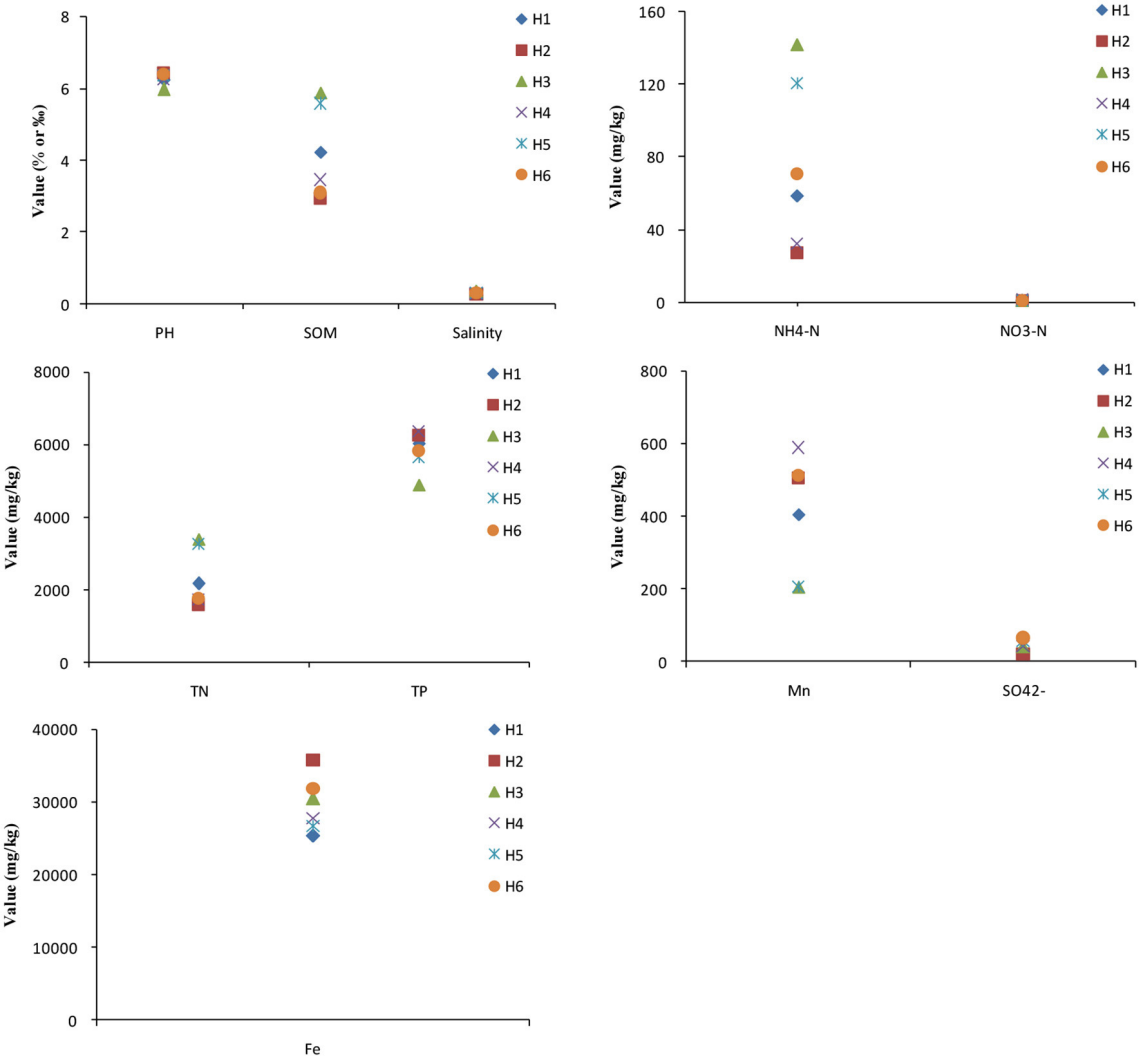
Physicochemical parameters of paddy wetland soils at different sampling sites.

The soil of paddy wetlands was weakly acidic and had low salinity for groundwater's irrigation at various depths, whereas the contents of SOM, TN, and TP were relatively high in the ranges of 2.93–5.90%, 1571.46–3397.90 mg/kg, and 4892.93–6347.79 mg/kg, respectively (Figure 2). The level of ${\text{NH}}_{4}^{+}$-N was high and that of ${\text{NO}}_{3}^{-}$-N was low. Fe, Mn and ${\text{SO}}_{4}^{2-}$ were abundant in paddy soil. In particular, SOM, ${\text{NH}}_{4}^{+}$-N, and TN had high levels in H3 and H5, indicating their good nutrient condition that was conducive to paddy growth and material transformation. Meanwhile, the low levels in H2 and H4 may be related to their soil structure and properties. The low ${\text{NO}}_{3}^{-}$-N level was consistent with high SOM amount, suggesting that a high SOM content in paddy soil could promote denitrification (Li et al., 2019). Mn had high levels in H2, H4, and H6; Fe was significantly enriched in H2, H3, and H6; and ${\text{SO}}_{4}^{2-}$ was high in H6. These findings implied that deep-groundwater's irrigation was beneficial to the enrichment of multiple electron acceptors. CA showed that salinity had a positive correlation with ${\text{NO}}_{3}^{-}$-N, and TN levels (*p* < 0.05; Supplementary Table 1), indicating that high salinity had inhibitory effects on denitrification (Wang et al., 2018a). SOM had a positive correlation with TN (*p* < 0.01), suggesting that SOM accumulating was beneficial to TN enriching. Mn was negatively correlated with SOM, ${\text{NO}}_{3}^{-}$-N, and TN, indicating that Mn was mainly affected by groundwater irrigation. No significant correlation was found among Fe, ${\text{SO}}_{4}^{2-}$, and other soil properties. In summary, SOM, TN, and TP were abundant in paddy soil; some differences were observed among the paddy soil samples for groundwater's irrigation at various depths; and deep-groundwater's irrigation could promote the accumulation of various electron acceptors.

### 3.2 Microbial community composition and difference

**Figure 3:**
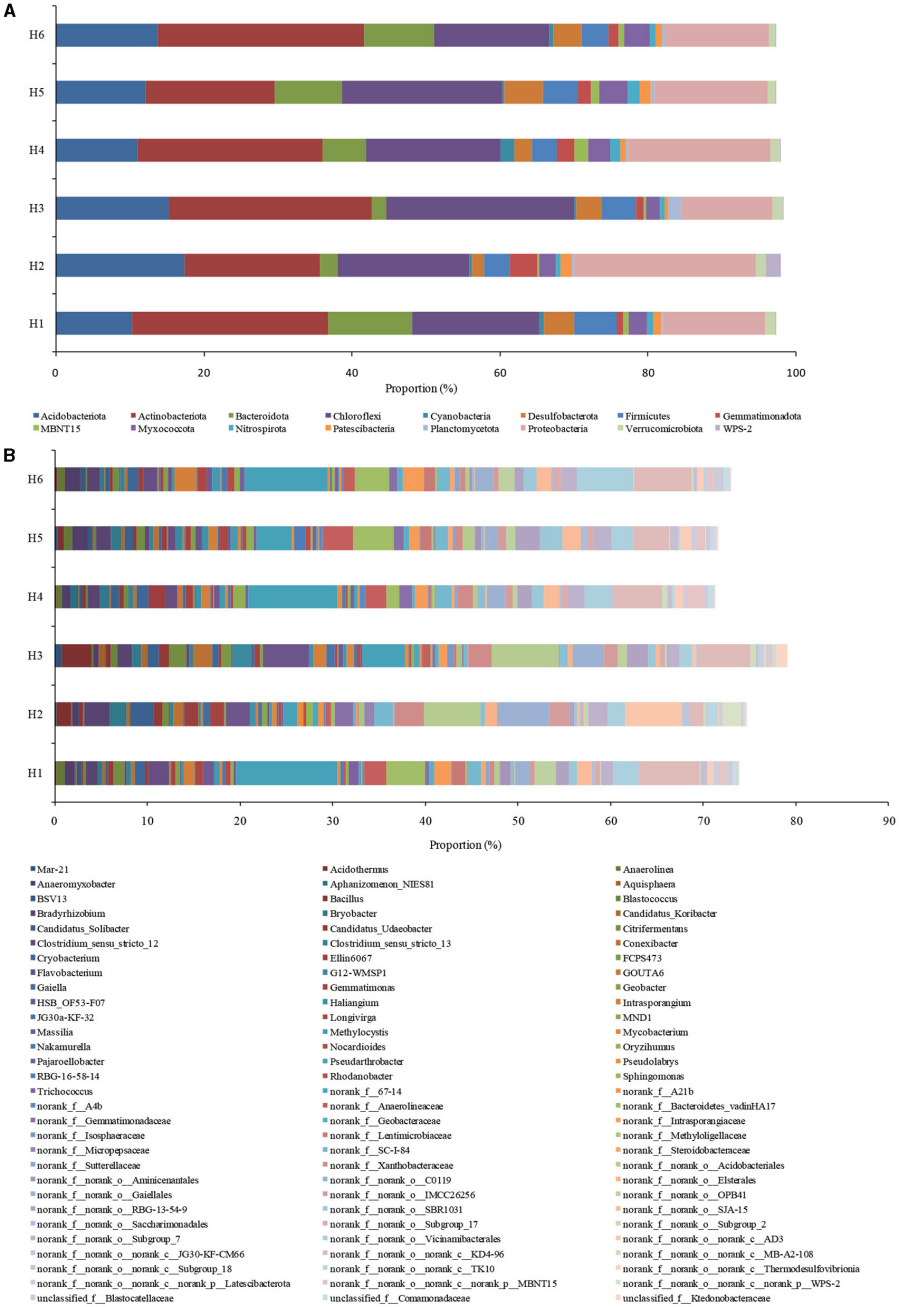
Taxonomic classification of bacterial community at **(A)** phylum and **(B)** genus levels. Less than 0.5% was not included.

The community of microorganisms under phylum- and genus- levels was shown in Figure 3. Acidobacteriota, Desulfobacterota, Actinobacteriota, Myxococcota, Bacteroidota, Firmicutes, Chloroflexi, Nitrospirota, Planctomycetota, and Proteobacteria were the important phyla with over 97% bacterial abundance (Figure 3A). Among them, Actinobacteriota, Chloroflexi, and Firmicutes had a correlation with organics degradation (He et al., 2018; Chi et al., 2021b), and Acidobacteriota, Bacteroidota, Nitrospirota, Planctomycetota, and Proteobacteria were connected with nitrogen cycling (Chi et al., 2021e). Desulfobacterota and Myxococcota were involved in sulfur and iron cycle (Langwig et al., 2022). Bacteroidota and Firmicutes were the most abundant in H1, and Acidobacteriota and Proteobacteria had the highest abundance in H2. The abundance of Acidobacteriota was lower in H1 than in H2-H6; this species played key roles in denitrification (Zhang et al., 2020). In addition, low abundance of Nitrospiota was consistent with the relatively anoxic environment of paddy wetlands. High abundance of Proteobacteria in paddy wetlands was relating to its diverse substrate-utilizing and environmental-adaptability (Bernhard et al., 2005). High abundances of Actinobacteriota and Chloroflexi were consistent with high SOM amount in the soil. Therefore, the phyla related to carbon and nitrogen cycling were widespread and abundant in paddy wetlands, and some phyla had high abundance under deep groundwater irrigation.

The dominant genera were MND1, *Sphingomonas, Ellin6067, Flavobacterium, Pseudolabrys, Rhodanobacter, Gemmatimonas, Anaeromyxobacter, Bacillus, Geobacter, Citrifermentans, Methylocystis, norank_f_Methyloligellaceae, Pseudarthrobacter*, and *Conexibacter* (Figure 3B). The abundances of nitrifying bacteria MND1 and *Sphingomonas* were relatively low, although ${\text{NH}}_{4}^{+}$-N level was high in paddy soil; the discrepancy may be related to the relatively anoxic environment of paddy wetlands (Tolar et al., 2020; Gao et al., 2021). High abundance of nitrifying bacteria *Ellin6067* was related to its diverse metabolic capabilities (such as nitrification and organic matter degradation) (Lezcano et al., 2017; Li et al., 2021b), and *Ellin6067* was abundant in deep groundwater irrigation. In addition, aerobic *Candidatus_Udaeobacter* was widely distributed in paddy soil but had low abundance, further indicating the relatively anoxic environment of paddy wetlands (Li et al., 2021a). The denitrifying bacteria *Flavobacterium* was widely distributed in paddy wetlands, with the highest abundance found under shallow groundwater irrigation (Pishgar et al., 2020). Meanwhile, *Pseudolabrys, Rhodanobacter*, and *Gemmatimonas* were abundant under deep groundwater irrigation (Van Den Heuvel et al., 2010; Green et al., 2012; Yan et al., 2019), indicating the diversity of denitrifying bacteria. As an aerobic denitrifier, *Gemmatimonas* could reduce N_2_O to N_2_ and decrease greenhouse gas emissions (Park et al., 2017). The high presence of denitrifying bacteria was consistent with the high SOM amount in the soil. The organic-degrading bacteria *Pseudarthrobacter* was abundant in all the samples, with the highest abundance found under shallow groundwater irrigation. *Conexibacter* had high abundance under deep groundwater irrigation. *Pseudarthrobacter* showed the organic matter degradation potential at low temperature (Zhang et al., 2016), and *Conexibacter* could decompose refractory organic matter (Liang et al., 2021). Iron-reducing bacteria *Geobacter* was abundant under shallow groundwater irrigation, and *Anaeromyxobacter, Bacillus*, and *Citrifermentans* were dominant under deep groundwater irrigation; all these genera had Fe-anammox potential (Chi et al., 2021a; Ottoni et al., 2022). Studies have shown that *Anaeromyxobacter* and *Geobacter* could participate in Fe-anammox (Zhou et al., 2016). Furthermore, *Geobacter* had dissimilar nitrate reduction potential and could drive dissimilatory nitrate reduction to ammonia (DNRA) using divalent iron, thus providing substrate for anammox (Finneran et al., 2002). Excitedly, *Intrasporangium* had chromium-reducing potential and was widely distributed in paddy wetlands (Yang et al., 2009). *norank_f_Gemmatimonadaceae* was abundant under deep groundwater irrigation and had good nitrate- and vanadium- reduction ability (Jia et al., 2019; Fei et al., 2022). The existence of these species could promote anammox. The methanotrophs *Methylocystis* and *norank_f_Methyloligellaceae* were widely distributed with low abundance, possibly due to the relatively anoxic environment of paddy wetlands. The presence of aerobic methane oxidation was beneficial to *in-situ* reduction of methane in paddy wetlands. Moreover, *HSB_OF53-F07* had nitrogen metabolism ability and diverse metabolic functions under aerobic/anoxic conditions (Wu et al., 2021). *Bradyrhizobium* with high abundance had nitrogen fixation function and could promote rice growth (Kaneko et al., 2002). *Candidatus_Solibacter* had high abundance under deep groundwater irrigation, which was consistent with the results in paddy soils under mine drainage irrigation and long-term fertilization (Wang et al., 2018b; Yu et al., 2019). *Candidatus_Solibacter* could degrade complex pollutants and outperform the competition in environmental filtration (Ward et al., 2009). *Cryobacterium* and *Trichococcus* had low temperature adaptations and may play important roles in material cycling (Pikuta et al., 2006; Teoh et al., 2021). Therefore, groundwater irrigation was beneficial to organics degradation, denitrification, and Fe-anammox. Meanwhile, nitrification and aerobic methane oxidation in paddy wetlands were relatively weak. Shallow groundwater irrigation increased organics degradation, but deep groundwater irrigation promoted the occurrence of Fe-anammox.

**Figure 4:**
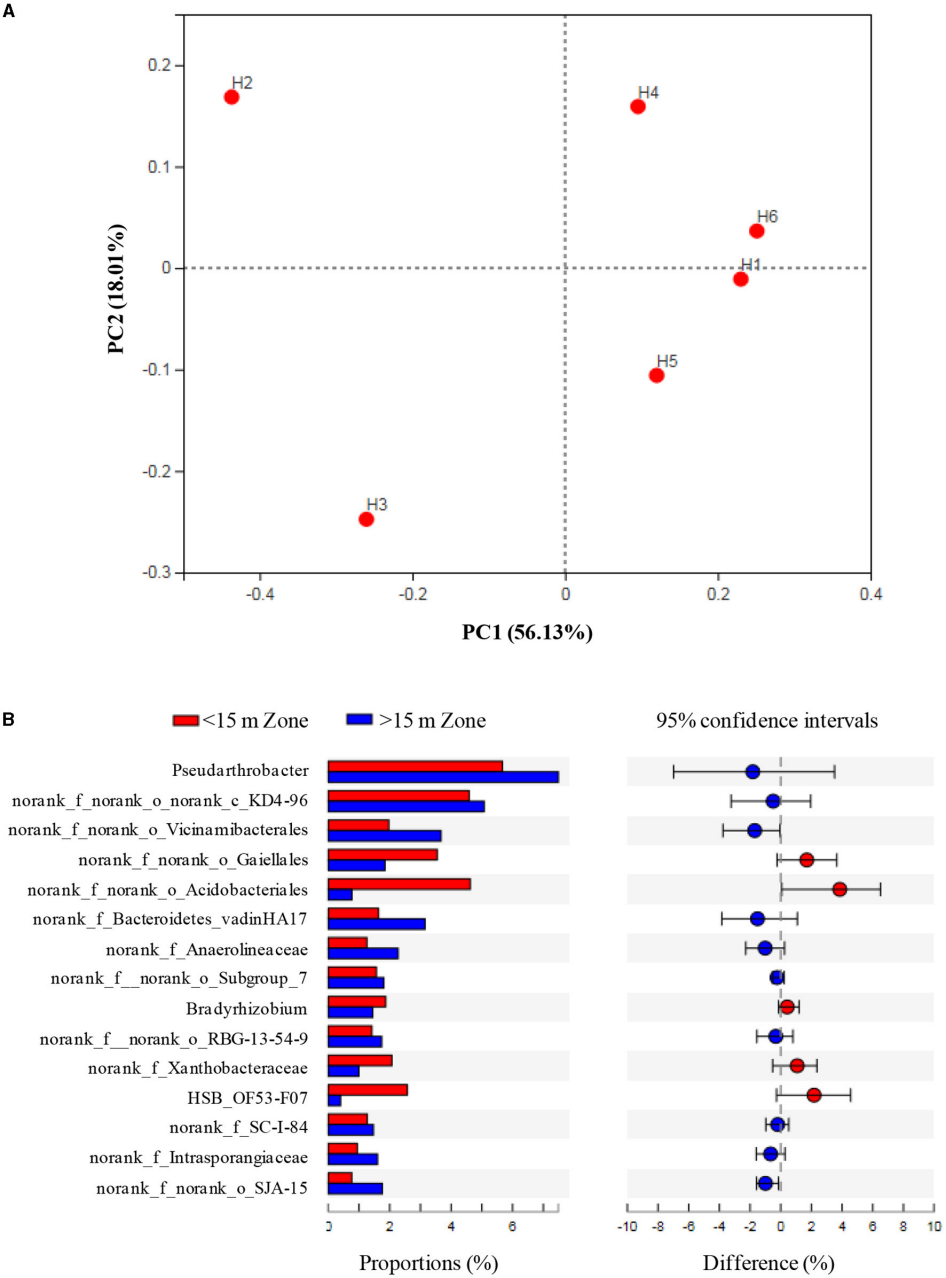
Principal coordinate analysis (PCoA) **(A)** and species differences **(B)** at different sampling zones.

PCoA suggested that the microbial communities had minimal differences under shallow and deep groundwater irrigation, but significantly differed with H2 and H3, which was related to high the SOM, ${\text{NH}}_{4}^{+}$-N, TN, and Fe levels (Figure 4A). The abundance of *Pseudarthrobacter* under groundwater irrigation (above 15 m) was higher than that in other irrigation zones (below 15 m) (*p* > 0.05) (Figure 4B). Meanwhile *Bradyrhizobium* and *HSB_OF53-F07* had high abundances in irrigation zones (below 15 m) (*p* > 0.05), which was consistent with high abundance of *Bradyrhizobium* and low count of *HSB_OF53-F07* and *Pseudarthrobacter* in H2 and H3.

### 3.3 Key variables influencing the community of microorganisms

**Figure 5:**
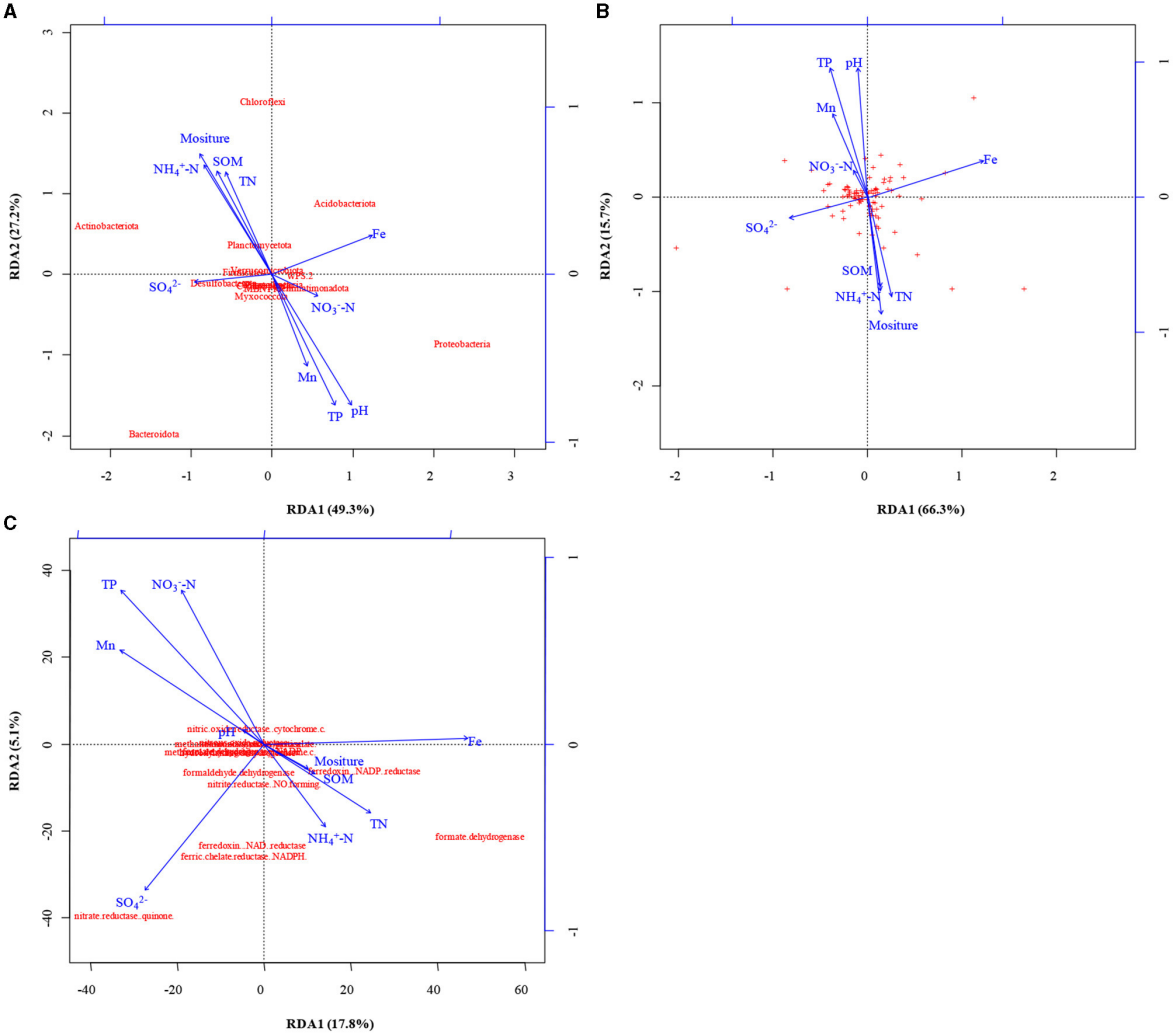
The results of redundancy analysis (RDA) between soil properties, microbial community at phyla **(A)** and genus **(B)** level, and functional genes **(C)**.

The Shannon's index was lower in shallow-groundwater's irrigation than in deep-groundwater's irrigation, indicating that deep-groundwater's irrigation with multiple electron acceptors was beneficial to the improvement of microbial diversity (Supplementary Figure 1). CA suggested that Acidobacteria was positively correlated with Fe (*p* < 0.01; Supplementary Table 2), expressing that Fe accumulation could promote Acidobacteria enrichment and facilitate denitrification. Chloroflexi had a positive correlation with SOM, salinity, moisture, ${\text{NH}}_{4}^{+}$-N, and TN, but negative correlation with TP, and Mn (p < 0.05), suggesting that SOM and TN accumulation was beneficial to Chloroflexi enrichment. Planctomycetes showed a significant positive correlation with salinity (*p* < 0.05). A previous study found that anammox bacteria Scalindua had favorable salinity adaptation (Zheng et al., 2016). RDA showed that moisture, pH, and TP were the major controlling factors at phylum level, and ${\text{NH}}_{4}^{+}$-N and multi-electron acceptors had significant effects at genus level (Figures 5A, B). Similarly, previous studies found that pH and moisture had crucial effects on the microbial community (Banning et al., 2011; Pan et al., 2021), and nutrients also could alter microbial composition (Francioli et al., 2018). Thus, moisture, pH, and TP had crucial effects in shaping microbial community under phylum- and genus-levels.

### 3.4 Function prediction of microorganisms

**Figure 6:**
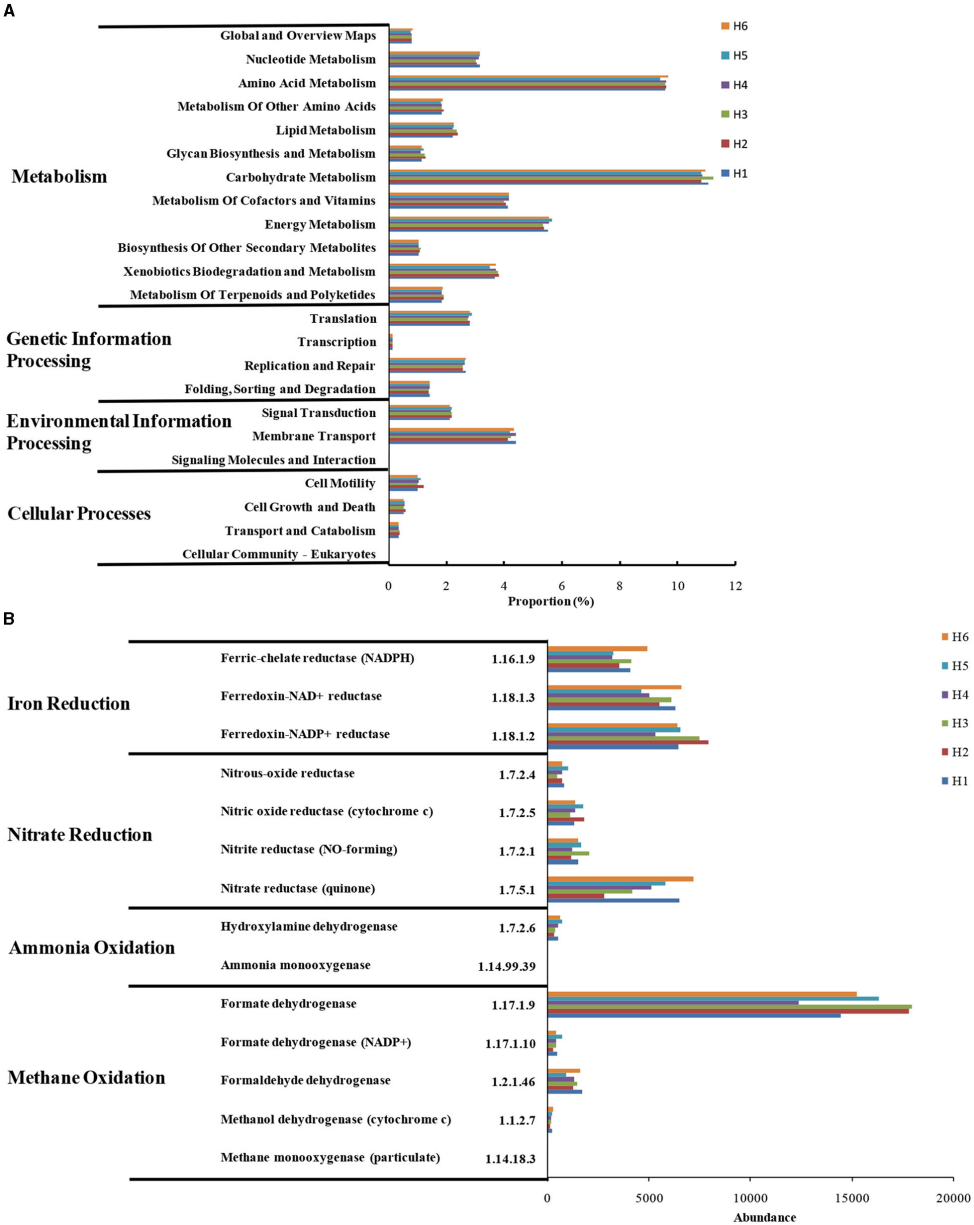
KEGG-predicted metabolic pathways **(A)** and functional genes **(B)** at different sampling sites.

Microbial functions were predicted by PICRUSt (Figure 6). Microorganisms were present in different metabolic pathways, such as metabolism, gene information's processing, environmental information's processing, and cellular processes (Li et al., 2018; Chi et al., 2021c); among which, their abundance in metabolism was the highest (Figure 6A). In particular, the genes involved in cell motility and growth/death were abundant, indicating the high microbial activity in paddy wetlands. High abundance of genes encoding carbohydrates, amino acids, and energy metabolism suggested the good material and energy metabolism of microorganisms. The gene encoding energy metabolism were abundant in H4-H6, and this finding was possibly related to the abundant supply of electron acceptors under deep groundwater irrigation. The expression of genes encoding xenobiotic biodegradation and terpenoids/polyketide metabolism was observed, indicating that microorganisms showed potential to metabolize exogenous substances. The genes related to signal transduction and membrane transport were highly abundant possibly due to the high nutrient levels in paddy wetlands. The repair-related gene was more expressed under shallow groundwater irrigation, indicating that microorganisms were alive to environmental variations. This findings were consistent with the low biodiversity in paddy wetlands.

These genes with regard to nitrogen and methane metabolism were analyzed as shown in Figure 6B. The nitrifying-related genes [EC:1.14.99.39 and EC:1.7.2.6] were abundant and highly expressed under deep groundwater irrigation, indicating that nitrification occurred in paddy wetlands. The nitrate-reductase gene [EC:1.7.5.1], which could promote denitrification and DNRA, was the most abundant (Chi et al., 2021f). High level of nitrite-reductase gene [EC:1.7.2.1] was beneficial to denitrification and anammox (Chi et al., 2021d). The abundance of denitrifying-related genes coincided with widespread denitrifying bacteria and low ${\text{NO}}_{3}^{-}$-N levels. Moreover, the genes related to iron reduction were highly expressed and highly abundant under deep groundwater irrigation. This finding indicated the existence of Fe-anammox in paddy wetlands, which was consistent with the enrichment of Fe-anammox bacteria. The genes encoding methane oxidation were abundant, especially formate dehydrogenase [EC:1.17.1.9], indicating the widespread occurrence of methane oxidation. In particular, the abundance of formate dehydrogenase [EC:1.17.1.9] was more abundant under deep groundwater's irrigation than that under shallow groundwater's irrigation, indicating that deep groundwater's irrigation favored its enrichment. The low level of methane-monooxygenase [EC:1.14.18.3] was consistent with the relatively anoxic environment of paddy wetlands. The complete methane oxidation-related genes was related to the widespread distribution of methanotrophs. Thus, deep groundwater irrigation could promote Fe-anammox, nitrification, and methane oxidation. Meanwhile, denitrification was ubiquitous under groundwater irrigation.

RDA indicated that electron acceptors were the main drivers of functional gene changes, and TN and TP had some effects (Figure 5C). Previous studies reported that nutrients expressed important effects on the functional genes (Chi et al., 2021b), and electron acceptors had a direct role (Li et al., 2022). CA showed that nitrite reductase (NO-forming) had a positive correlation with SOM, salinity, moisture, ${\text{NH}}_{4}^{+}$-N, and TN (*p* < 0.01 or *p* < 0.05) but negative correlation with TP, and Mn (*p* < 0.01 or *p* < 0.05; Table 1), indicating that SOM and TN accumulation was favorable for denitrification. Furthermore, nitrite reductase (NO-forming) showed a positive correlation with ${\text{NH}}_{4}^{+}$-N (*p* < 0.01), suggesting the possibility of anammox (Li et al., 2019). In addition, ${\text{NH}}_{4}^{+}$-N had a positive correlation with ammonia monooxygenase, and hydroxylamine dehydrogenase (*p* > 0.05), implying that ${\text{NH}}_{4}^{+}$-N accumulation could promote nitrification. The genes with respect to iron reduction [ferredoxin–NADP+ reductase, ferredoxin–NAD+ reductase, and ferric-chelate reductase (NADPH)] were positively correlated with ${\text{NH}}_{4}^{+}$-N, and Fe (*p* > 0.05), indicating the existence of Fe-anammox of paddy wetlands.

**Table 1:** Correlation coefficient matrix between functional genes and physicochemical properties.

	**pH**	**SOM**	**Salinity**	**Moisture**	**${\text{NH}}_{4}^{+}$-N**	**${\text{NO}}_{3}^{-}$-N**	**TN**	**TP**	**Fe**	**Mn**	** ${\text{SO}}_{4}^{2-}$ **
Methane monooxygenase (particulate)	−0.119	0.575	0.215	0.327	0.398	0.251	0.579	−0.218	−0.159	−0.517	0.032
Methanol dehydrogenase (cytochrome c)	0.278	0.083	0.030	−0.150	0.289	−0.491	0.129	−0.119	−0.391	−0.153	0.772
Formaldehyde dehydrogenase	−0.295	−0.291	0.113	0.067	−0.195	−0.197	−0.333	−0.023	−0.020	0.318	−0.133
Formate dehydrogenase (NADP+)	0.016	0.643	0.262	0.310	0.602	−0.311	0.647	−0.304	−0.702	−0.636	0.566
Formate dehydrogenase	−0.104	0.366	0.374	0.276	0.491	−0.657	0.454	−0.593	0.576	−0.587	−0.181
Ammonia monooxygenase	−0.119	0.575	0.215	0.327	0.398	0.251	0.579	−0.218	−0.159	−0.517	0.032
Hydroxylamine dehydrogenase	0.327	0.146	−0.094	−0.162	0.208	−0.116	0.161	0.073	−0.613	−0.124	0.759
Nitrate reductase (quinone)	0.116	0.004	0.014	−0.089	0.128	−0.243	−0.001	0.012	−0.588	0.030	0.668
Nitrite reductase (NO-forming)	−0.719	0.860^*^	0.958^**^	0.831^*^	0.958^**^	−0.559	0.887^*^	−0.981^**^	−0.215	−0.857^*^	0.353
Nitric oxide reductase (cytochrome c)	0.830^*^	−0.290	−0.685	−0.597	−0.331	−0.016	−0.237	0.494	0.309	0.109	−0.158
Nitrous-oxide reductase	0.637	0.011	−0.492	−0.333	−0.097	−0.028	0.005	0.420	−0.483	−0.059	0.244
Ferredoxin—NADP+reductase	−0.037	0.156	0.187	0.167	0.232	−0.581	0.221	−0.388	0.657	−0.383	−0.448
Ferredoxin—NAD+reductase	−0.235	−0.209	0.229	0.057	0.012	−0.491	−0.194	−0.248	0.216	0.136	0.007
Ferric-chelate reductase (NADPH)	−0.100	−0.179	0.243	−0.049	0.139	−0.574	−0.124	−0.314	0.228	0.078	0.332

^*^p < 0.05 level significant correlation. ^**^p < 0.01 level significant correlation.

## 4 Conclusions

This study illustrated the potential effects of groundwater's irrigation on the community and function of microorganism in paddy wetlands. Deep-groundwater's irrigation favored the accumulation of multi-electron acceptors. Groundwater irrigation was beneficial to organics degradation, denitrification, and Fe-anammox, but nitrification and aerobic methane oxidation were relatively weak in paddy wetlands. Moisture, pH, and TP played vital roles for microbial communities shaping. Microorganisms were highly sensitive to environmental changes under shallow-groundwater's irrigation, and deep-groundwater's irrigation favored Fe-anammox, nitrification, and methane oxidation. Denitrification was widespread under groundwater's irrigation. The findings offer new ideas for non-point source pollution control and greenhouse gas reduction in paddy wetlands.

## Data availability statement

The original contributions presented in the study are included in the article/Supplementary material, further inquiries can be directed to the corresponding author.

## Author contributions

HL: Writing—original draft. AS: Writing—review & editing. LQ: Writing—review & editing. SL: Writing—review & editing. ZC: Conceptualization, Funding acquisition, Supervision, Writing—review & editing.
