# Supplementary material for: Deep groundwater irrigation altered microbial community and increased anammox and methane oxidation in paddy wetlands of Sanjiang Plain, China
Source: Front Microbiol. 2024 Feb 21;15:1354279. doi: 10.3389/fmicb.2024.1354279 (PMC10915080; doi:10.3389/fmicb.2024.1354279)
Supplement: Supplementary file 1 [file Data_Sheet_1.docx]

Fig.S1 Shannon indices of different sampling sites.

Table S1 Correlation coefficient matrix of soil properties.

|  | pH | SOM | Salinity | Moisture | NO_3_^-^-N | NH_4_^+^-N | TN | TP | Fe | Mn | SO_4_^2-^ |
| --- | --- | --- | --- | --- | --- | --- | --- | --- | --- | --- | --- |
| pH | 1 |  |  |  |  |  |  |  |  |  |  |
| SOM | -0.648 | 1 |  |  |  |  |  |  |  |  |  |
| Salinity | -0.871* | 0.822* | 1 |  |  |  |  |  |  |  |  |
| Moisture | -0.898* | 0.888* | 0.884* | 1 |  |  |  |  |  |  |  |
| NO_3_^-^-N | -0.566 | 0.912* | 0.881* | 0.755 | 1 |  |  |  |  |  |  |
| NH_4_^+^-N | -0.023 | -0.342 | -0.328 | -0.218 | -0.540 | 1 |  |  |  |  |  |
| TN | -0.589 | 0.988** | 0.829* | 0.835* | 0.954** | -0.415 | 1 |  |  |  |  |
| TP | 0.699 | -0.810 | -0.952** | -0.782 | -0.940** | 0.550 | -0.855* | 1 |  |  |  |
| Fe | 0.289 | -0.469 | -0.203 | -0.438 | -0.247 | -0.117 | -0.369 | 0.035 | 1 |  |  |
| Mn | 0.471 | -0.949** | -0.747 | -0.776 | -0.926** | 0.581 | -0.971** | 0.830* | 0.287 | 1 |  |
| SO_4_^2-^ | 0.065 | 0.196 | 0.270 | -0.070 | 0.458 | -0.170 | 0.274 | -0.318 | -0.207 | -0.181 | 1 |

* p<0.05 level significant correlation.

* *p<0.01 level significant correlation.

Table S2 Correlation coefficient matrix between dominant phyla and physicochemical properties.

|  | pH | SOM | Salinity | Moisture | NH_4_^+^-N | NO_3_^-^-N | TN | TP | Fe | Mn | SO_4_^2-^ |
| --- | --- | --- | --- | --- | --- | --- | --- | --- | --- | --- | --- |
| Acidobacteria | 0.100 | -0.144 | 0.046 | -0.149 | 0.039 | -0.272 | -0.043 | -0.234 | 0.936** | -0.050 | -0.244 |
| Actinobacteria | -0.576 | -0.033 | 0.433 | 0.282 | 0.087 | -0.003 | -0.070 | -0.271 | -0.184 | 0.171 | 0.213 |
| Bacteroidetes | 0.333 | -0.057 | -0.253 | -0.183 | -0.055 | -0.246 | -0.088 | 0.256 | -0.657 | 0.052 | 0.388 |
| Chloroflexi | -0.716 | 0.888* | 0.833* | 0.841* | 0.818* | -0.145 | 0.893* | -0.816* | -0.117 | -0.828* | 0.041 |
| Desulfobacterota | -0.079 | 0.620 | 0.383 | 0.364 | 0.665 | -0.546 | 0.631 | -0.439 | -0.663 | -0.644 | 0.612 |
| Firmicutes | -0.389 | 0.630 | 0.420 | 0.666 | 0.483 | -0.562 | 0.567 | -0.409 | -0.621 | -0.645 | -0.153 |
| Myxococcota | 0.567 | -0.033 | -0.278 | -0.423 | 0.084 | -0.029 | 0.022 | 0.192 | -0.301 | 0.002 | 0.770 |
| Nitrospirota | 0.231 | 0.222 | -0.187 | -0.063 | 0.057 | 0.402 | 0.189 | 0.259 | -0.655 | -0.092 | 0.402 |
| Planctomycetes | -0.796 | 0.692 | 0.906* | 0.786 | 0.759 | -0.327 | 0.719 | -0.912* | 0.170 | -0.681 | 0.008 |
| Proteobacteria | 0.581 | -0.637 | -0.750 | -0.641 | -0.738 | 0.480 | -0.629 | 0.704 | 0.559 | 0.576 | -0.544 |

* p<0.05 level significant correlation.

* *p<0.01 level significant correlation.
